# Building Public Health Data Dashboards: Tutorial Playbook

**DOI:** 10.2196/83157

**Published:** 2026-04-09

**Authors:** Elwin Wu, Raymond Balise, Benjamin Katz, Daniel Harris, Matthew Bullard, Naleef Fareed, Marc Larochelle, Jennifer Villani

**Affiliations:** 1 Social Intervention Group, Columbia University School of Social Work, 1255 Amsterdam Avenue, New York, NY, 10027, United States, 1 917-439-7391; 2 Division of Biostatistics and Bioinformatics, Department of Public Health Sciences, University of Miami, Miami, FL, United States; 3 Institute for Implementation Science in Population Health, City University of New York (CUNY), New York, NY, United States; 4 Institute for Biomedical Informatics, University of Kentucky, Lexington, KY, United States; 5 Center for Health Data Science, Boston University School of Public Health, Boston, MA, United States; 6 CATALYST – The Center for the Advancement of Team Science, Analytics, and Systems Thinking, College of Medicine, The Ohio State University, Columbus, OH, United States; 7 Clinical Addiction Research and Education Unit, Section of General Internal Medicine, Department of Medicine, Boston University School of Medicine and Boston Medical Center, Boston, MA, United States; 8 National Institute of Drug Abuse, National Institutes of Health, Bethesda, MD, United States

**Keywords:** dashboard systems, data visualization, public health, opioid epidemic, drug overdose

## Abstract

Public health data dashboards have substantial potential to improve transparency, understanding, and decision-making at multiple levels, from individuals to public health practitioners and policymakers. However, creating effective dashboards presents many challenges. In this case-based tutorial on public health dashboard development, we share lessons learned from our experience developing data dashboards for the HEALing Communities Study (HCS), a National Institutes of Health (NIH)–funded, community-engaged intervention to deploy evidence-based practices to reduce opioid overdose deaths in 67 communities across 4 states. We present key decision points dashboard teams must address, along with the major considerations and trade-offs that shaped our approach. First, we describe core considerations of the who, what, why, where, when, and how of data dashboard development. Second, we outline steps in data curation, including the identification of key metrics and potential data sources and developing processes to acquire the data. Third, we discuss practical aspects of developing data visualizations that can effectively communicate key messages to the end users of interest. Fourth, we describe the infrastructure considerations to host and publish data dashboards. And finally, we discuss maintenance and sustainability of the dashboard. While the material can be read sequentially as a step-by-step guide, we refer to this resource as a “playbook” because readers may engage with specific domains in a random-access fashion, that is, based on their specific needs and/or starting point rather than a fixed sequence. The information, supplemental materials, and resources will assist individuals and organizations seeking to build data dashboards by fostering context-sensitive evaluation of design and implementation choices to realize the promise of data-driven decision-making.

## Introduction

Automobile instrument panels are perhaps the most familiar type of dashboard. They combine analog and digital visualizations—for example, gauges, counters, and icons—to help drivers monitor key aspects of their vehicle, including fuel levels, speed, engine temperature, and mileage. Warning indicators alert drivers to critical issues like low oil, low tire pressure, or an unfastened seatbelt. Designed for instant readability, these dashboards prioritize essential information, with standardized warning lights and speedometers placed centrally to capture the driver’s immediate attention.

Public health data dashboards function much like automobile dashboards; both use data visualizations to communicate critical information and support decision-making. By consolidating valid and useful epidemiological metrics and other public health data, these dashboards enhance awareness, facilitate monitoring, and drive informed action. Their value has been especially evident during public health crises, such as the COVID-19 pandemic and the opioid overdose epidemic, where real-time data have played a crucial role in response efforts [1-3].

Despite an influx of public health data, challenges and barriers have emerged with regard to the effective and efficient communication of these data to a broad range of individuals. Issues critical to the uptake and use of public health data dashboards include a design that contains the most useful data, usability, cognitive load, implementation and evaluation, and sustainability [4]. Studies on data visualizations and dashboards have discussed notable challenges that pertain to these issues, including lack of rigorous scientific methodology, superficial or incorrect application of design principles, data literacy of end users, and limited scope in the representation of end user input [5-7].

Within this context, our research team engaged in the development of data-driven dashboards for a large, multisite cluster-randomized pragmatic trial. The HEALing Communities Study (HCS) was a community-based cluster-randomized trial aimed at addressing the opioid crisis in communities with high opioid overdose death rates. The National Institute on Drug Abuse (NIDA), in partnership with the Substance Abuse and Mental Health Services Administration (SAMSHA), funded the study in April 2019 to test the immediate impact of the implementation of an integrated set of evidence-based practices across health care, behavioral health, justice, and other community-based settings to prevent opioid overdose deaths and treat opioid use disorder [8]. Among the 4 states (“study sites”) involved in the HCS, communities (n=67) were randomized to receive the intervention first or be in the wait-list control group. In each community, a community coalition was established to provide local leadership and data-driven decision-making for the intervention [9]. Data dashboards—to support data-driven decision-making [10]—were co-created with coalition members to provide visualizations of key metrics on opioid overdoses and treatment for opioid use disorder within a community [3]. The coalition stakeholders (1) used data to define their local opioid situation, including prevention and treatment gaps and resources; (2) developed a data-driven action plan for implementing evidence-based practices linked to local needs; and (3) after deploying the action plan, they used data dashboards to monitor progress and adjust as needed.

Eighteen months after deployment, we conducted a usability evaluation of the HCS dashboards using a mixed methods approach that involved quantitative feedback from the System Usability Survey (SUS) [11] and qualitative interviews guided by the Technology Adoption Framework [12]. The SUS scores indicated the end users found HCS dashboards to be acceptable for usability; for example, the overall grand mean SUS score from 62 users of the HCS dashboards (39% response rate) was 73 (SD 4.6), which falls between the 70th and 80th percentile of SUS responses and is comparable to scores reported for widely used tools such as Microsoft Word and PowerPoint (Microsoft Corp) [13]. The interview results indicated positive feedback around dashboard usability and intention to use from the end users sampled.

The considerations described in this case-based tutorial playbook are grounded in our prior work in the development and evaluation of HCS data dashboards. Although some aspects of HCS dashboard development reflected consensus decisions or externally imposed requirements (eg, minimum required metrics and National Institutes of Health [NIH] funding conditions), the purpose of this tutorial playbook is not to present consensus guidance or a prescriptive methodology. Rather, we focus on identifying key decision points that arise during public health dashboard development and the attendant major considerations and trade-offs that shape how those decisions are approached. By emphasizing decision points rather than prescriptive solutions, this tutorial playbook supports adaptation across different public health settings. The manuscript is organized along a typical dashboard development lifecycle, from conceptual considerations through postlaunch revision. While this can be used as a step-by-step guide, dashboard development is often nonlinear; thus, we refer to this document as a “playbook” to indicate that readers can refer to specific sections as dictated by their context, starting point, and immediate needs at a given time.

## Public Health Dashboards: Key Tasks

Based on lessons learned from the HCS, we provide this playbook for creating public health data dashboards. While HCS dashboards focused exclusively on opioid use disorder and overdose-related metrics for each community, the information and considerations provided here can be applied to myriad public health issues. Consistent with the case-based foundation of this manuscript, the core considerations and domains are presented as guiding questions and decision points rather than as required artifacts (ie, tools or steps that are applicable across all contexts, such as accessibility review checklists, compliance sign-off documentation, and articulated user personas), success criteria, or approval gates that are likely to vary substantially both qualitatively and quantitatively across contexts.

**Figure 1.**
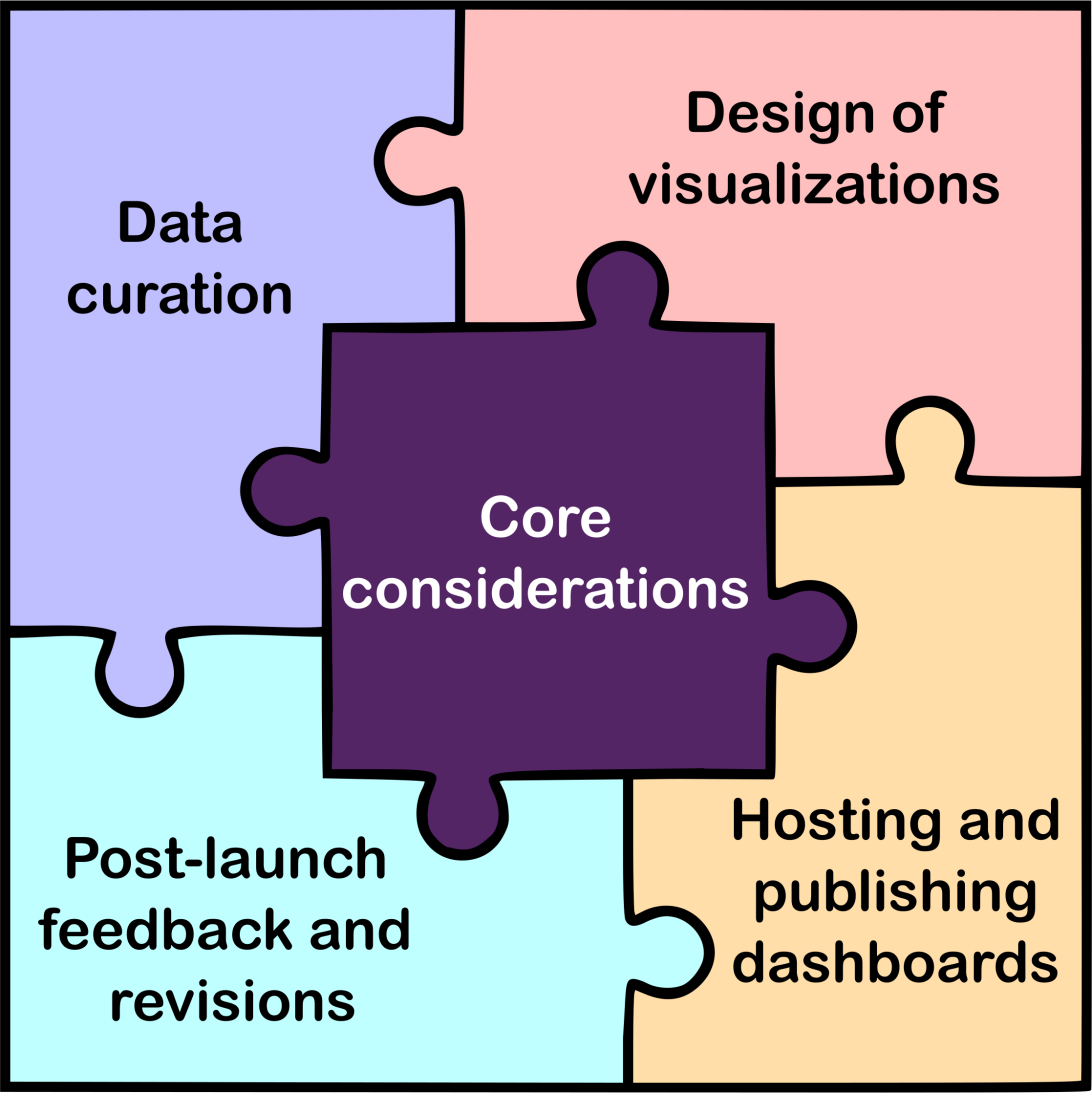
Overview of the playbook.

Each section is a conceptual domain of questions that help guide dashboard developers as they design and deploy a dashboard. For purposes of this tutorial playbook, we organize these along a prototypical dashboard lifecycle, starting with (1) core considerations and then progressing through (2) data curation, (3) design of visualizations, (4) hosting and publishing dashboards, and (5) postlaunch feedback and revision. However, as depicted in Figure 1, the process is neither linear nor discrete. Each section in the playbook is equally important, and the interrelationships and/or interplay among domains require developers to revisit decisions across domains over time.

### Core Considerations

A useful framework for guiding dashboard development at every stage is based on the classic journalistic “Five Ws and One H” approach: who, what, why, where, when, and how. These questions help ensure a structured and comprehensive approach to designing, deploying, and maintaining public health data dashboards (Textbox 1). By addressing these key decision-making topics, dashboard developers can ensure a holistic approach to planning, designing, and deploying effective dashboards. The core decision-making topics manifest throughout the key domains involved in dashboard development and publishing that are described in the ensuing sections of this playbook.

Textbox 1. **Core considerations.**

The “Five Ws and One H” approach:

- Who is the target audience (ie, end users)?
- What do the end users want to be able to learn or infer from the data?
- Why are dashboards a useful or the optimal tool to meet the need?
- Where will the dashboard be published and/or made available?
- When do the dashboards need to be published, updated, etc?
- How will we actually build and publish the dashboards?

#### Who: Understanding the End Users

Identifying the target audience—or end users—is essential. Dashboard developers must assess and tailor to their users’ technical expertise, data literacy, professional roles, and specific needs. Workflows also shape dashboard requirements; for instance, some users may need real-time monitoring, while others might only check a dashboard weekly, monthly, or quarterly.

#### What: Defining the Dashboard’s Purpose

Dashboards should be designed to answer key questions for end users efficiently. Developers must determine, ”What information do users need? How can the data be presented to support decision-making?” Ideally, visualizations should allow users to grasp insights quickly with minimal cognitive effort, delivering essential information “at a glance.”

#### Why: Establishing the Dashboard’s Core Objectives

This question prompts developers to define the overarching goals of the dashboard. What key messages or insights should be communicated? What operational or policy decisions should the dashboard support? Design choices—such as whether to use graphical summaries or textual explanations—should align with these objectives to maximize clarity and usability.

#### When: Considering the Timeline for Development and Updates

Developers must plan for the time required to acquire and integrate data, design and test the dashboard, and manage its lifecycle post launch. This includes establishing a schedule for updates, revisions, and long-term sustainability.

#### Where: Ensuring Availability and Visibility

The dashboard’s availability is a critical consideration. Developers must decide whether it should be hosted on a public website or restricted to an internal platform. If public, should it be optimized for search engines, or should access be controlled? Additionally, dashboards should be designed to function seamlessly across different devices—desktops, laptops, tablets, and smartphones—each of which has unique constraints related to screen size, processing power, and internet connectivity. In some cases, a dedicated app or embedded dashboards within an existing platform may enhance usability.

#### How: Assessing Resources and Infrastructure

Successful dashboards require technological and human resources. Developers must evaluate factors such as cybersecurity, data storage, processing capabilities, and integration with external data sources. Managing these elements effectively ensures the dashboard remains functional, secure, and scalable over time.

In addition, governance is an important consideration, as decisions about data access, processing, and publication are often shaped by legal, contractual, and institutional constraints. In practice, dashboard teams need to address how data use agreements (DUAs), applicable privacy regulations (eg, the Health Insurance Portability and Accountability Act [HIPAA]), and data suppression rules affect what data can be ingested, how it can or needs to be transformed and displayed, and with whom it can be shared. These constraints often extend beyond technical implementation to influence workflows, timelines, and responsibilities across the data lifecycle, from data acquisition and quality assurance through publication, revision, and eventual retirement.

An information architecture diagram can help document how data move through a system, from source data through transformation and storage to visualization, access control, and monitoring. Such diagrams may be useful for identifying or clarifying the human and technical resources involved, how those resources are organized, key dependencies, and where governance and security considerations arise, as well as for supporting communication among technical and nontechnical stakeholders involved in dashboard development.

### Data Curation

Data curation involves identifying desired metrics, sourcing available data, and establishing procedures to acquire or “ingest” the data (Textbox 2). This process is foundational to building a reliable and actionable public health dashboard. Desired metrics—shaped primarily by the who, what, and why—typically are the primary outcome and key epidemiological characteristics and/or targets of prevention and intervention efforts (eg, incidence or prevalence of a disease or phenomenon of interest). Other data that are often desired include secondary outcomes, key drivers of the outcomes of interest, and data that allow for subgroup characterization (eg, age, sex, race, ethnicity, geography).

Textbox 2. **Decision points for data curation.**

The following questions outline key decision points to consider when planning and implementing data curation processes for dashboards and other data-driven systems:

- What are the desired metrics and visualizations?
- What data are available?
- What are the acquisition requirements (eg, data use agreements and security needs) and associated costs for infrastructure and staffing?
- What are ways to address data quality and refresh discipline?
- How do these requirements match available or planned resources and funding?
- Based on all of the above, what are the relative priorities of potential metrics and visualizations?

Many metrics require calculations that integrate multiple data points. For example, prevalence is derived from case counts relative to a denominator—typically population size—which may also need stratification by demographic variables. Similarly, spatial visualizations require both temporal and geographic details for each case. Thus, desired metrics may involve multidimensional databases or combining and calculating across different data sources. Regardless of the exact metric, the most effective dashboards ensure that such data are not only accurate but also actionable (eg, lag between data collection and reporting does not undermine relevance for decision-making).

In identifying available data, several potential sources should be considered. Administrative data may be collected and made available from government agencies and initiatives (eg, vital statistics, Medicaid, or prescription drug monitoring programs) as well as service providers (eg, health care providers and social service agencies). Data may be collected and made available from research studies, program evaluations, population-level surveys (eg, the National Survey on Drug Use and Health and the Behavioral Risk Factor Surveillance System), and contracted third parties (eg, HCS used data from IQVIA and Millenium Health). Many data repositories also make data available (eg, Centers for Disease Control and Prevention [CDC] WONDER and the National Notifiable Diseases Surveillance System). Existing dashboards and other surveillance or monitoring systems may provide data directly or, when permitted, be incorporated into planned dashboards via embed codes that display existing visualizations without transferring the underlying data, or through scraping data from existing visualizations in accordance with applicable terms of use for local processing into a new visualization.

Constraints often govern the use and visualization of data, shaping how dashboards can present and share information. Data providers may impose restrictions on who can access specific datasets, limiting or prohibiting certain audiences from viewing them. These conditions are typically outlined in a DUA, which defines the expectations, requirements, and responsibilities between data providers and those developing and publishing the dashboard. A DUA specifies what data are being shared, the procedures for data transmission, storage, and publication, and any restrictions on visualization. These may include requirements for attribution, contextual definitions, or limitations on further distribution. Additionally, government regulations and institutional policies often impose further constraints, such as suppression rules designed to protect privacy and confidentiality. These rules dictate how data can be displayed, ensuring that sensitive or identifiable information remains secure while maintaining the integrity and utility of the dashboard. Other privacy-preserving computation approaches (eg, secure multiparty computation and federated approaches) may also be relevant, particularly for analyses that span data across multiple organizations or jurisdictions, although such approaches can involve greater technical complexity and coordination.

Within these constraints, data quality and data refresh are ongoing considerations in data curation. HCS teams regularly examined and tracked data quality dimensions such as completeness, timeliness and lag, internal consistency, and stability over time [14]. Dashboard designers may want to display refresh schedules or lag indicators, and the use of tools such as data quality scorecards (eg, to assess whether data meet thresholds indicating sufficient quality or warrant caution) may be useful for standardization, monitoring, and quality assurance. We note, however, that appropriate thresholds and related decisions depend heavily on context, including data source characteristics, suppression requirements, the potential clinical or policy impact of over- or misinterpretation, and intended dashboard use. Those are some of the aspects that allow for tailoring of data quality and refresh discipline (ie, the exact metrics, thresholds, or automation approaches for detecting problems stemming from lower quality or lag in data).

Data ingestion refers to the process of transferring data from its source to the recipient responsible for building the dashboard. This transfer can occur through various methods, including pushing, where the data provider actively transmits the data; pulling, where the recipient requests and retrieves the data; and scraping, where data are electronically extracted from existing documents or publications when permitted and in accordance with publishers’ terms of use. In many cases, ingestion also involves preliminary data processing to ensure compatibility with dashboard visualization and storage. This preprocessing follows an extract, transform, and load workflow, which may include data cleaning (such as removing disallowed values or correcting inconsistencies), transforming variables (such as calculating rates from raw counts or aggregating data across time periods), and restructuring datasets to align with the platform used for visualization. The efficiency and reliability of data ingestion improve with greater automation. For example, some data sources provide an application programming interface, a standardized set of protocols that allow software to directly request and retrieve data from the source, subject to authorization and applicable restrictions on use, storage, or display. However, implementing automation requires investment in human resources for design, programming, and ongoing maintenance as data systems evolve or become obsolete. Before establishing a data ingestion plan, it is essential to assess the capabilities of the existing cyberinfrastructure. If these resources are inadequate, the costs—whether in terms of personnel, hardware, or development time—must be factored into planning. When budgets and timelines are constrained, trade-offs may become necessary, potentially limiting the scope of data sources or the complexity of visualizations that can be supported.

Selection of data to be ingested for visualizations often cycles back and forth among desired versus available data, utility of data, and resources needed to transform the data into visualizations as well as maintain the cyberinfrastructure that hosts and publishes the dashboards. Desired metrics usually drive a targeted search for the relevant data needed to calculate and display the metric. Conversely, an examination of available data may prompt the desire for additional data (eg, disaggregated into subgroups such as race or ethnicity or age group, subtypes of substances, or variants of infectious agents; greater specificity with respect to time or geography). Given that resources do not allow for every possible visualization—nor is it advisable from a health communications perspective to provide vast quantities of information all at once—the full menu of potential visualizations should be prioritized for creation and inclusion in the dashboards. This decision-making process is about the appropriate or, ideally, optimal balance among data quality, utility of the metric, and costs or resources available to curate the data and publish the dashboards.

In public health, researchers, policymakers, and practitioners play a central role in determining key metrics and are typically considered the primary end users of data dashboards. The HCS operated under the principle that community members bring invaluable perspectives on the relevance, utility, and limitations of different data and metrics. Toward this end, HCS researchers developed the Community-Engaged Data Science model to assist data scientists working with communities in identifying and integrating relevant data for making informed decisions [15]. For data dashboard development, this ethos translated into a co-creation approach that sought to ensure that those affected by public health decisions, whether or not they hold formal decision-making roles, were actively involved in identifying and prioritizing the metrics and visualizations that would shape the dashboard. Engaging community members from the outset of the project fostered greater relevance, usability, and impact, making the dashboard a more effective tool for addressing the overdose crisis and other real-world public health challenges.

### Design of Visualizations

Ideally, the team-building dashboards include experts in human-centered design and graphical design who can work with the consumers of the displays (Textbox 3). A starting point is understanding the wants and needs of the end users. Interviews of end users can capture both what they want to know at a glance and what inferences and conclusions they would like to support (eg, trends in the data over time). During the initial development, focus groups and public meetings allow design teams to identify the types of people that will be using the dashboard. Understanding their data literacy—the ability to read and understand data and data visualizations—is paramount. For example, knowing that a dashboard will be used by public health practitioners would suggest that terms like “incidence” and “prevalence” can be used unabashedly, but if the same dashboard will be used by people without any background in statistics or epidemiology, avoiding jargon and using plain language is preferred to avoid confusing and alienating the audience. In the context of designing public health dashboards, it is critical to identify all end user groups and then develop an outreach plan to make sure that all constituents are assessed, rather than just the vocal and/or immediately available audience who may first provide information.

Textbox 3. **Decision points for the design of visualizations.**

The following questions highlight key considerations for designing effective visualizations that support user understanding and informed decision-making:

- How can we engage and elicit feedback from all end users?
- How can the data be transformed and/or presented so the end users can grasp the desired point at a glance?
- What additional visual elements will aid in comprehension or reduce misinterpretation?
- How can specific design elements be tweaked or refined to take advantage of how humans process information?
- What ancillary information is needed or helpful to the user?
- How can the dashboard interface allow the user to access such information without distracting from the main visualization?

**Figure 2.**
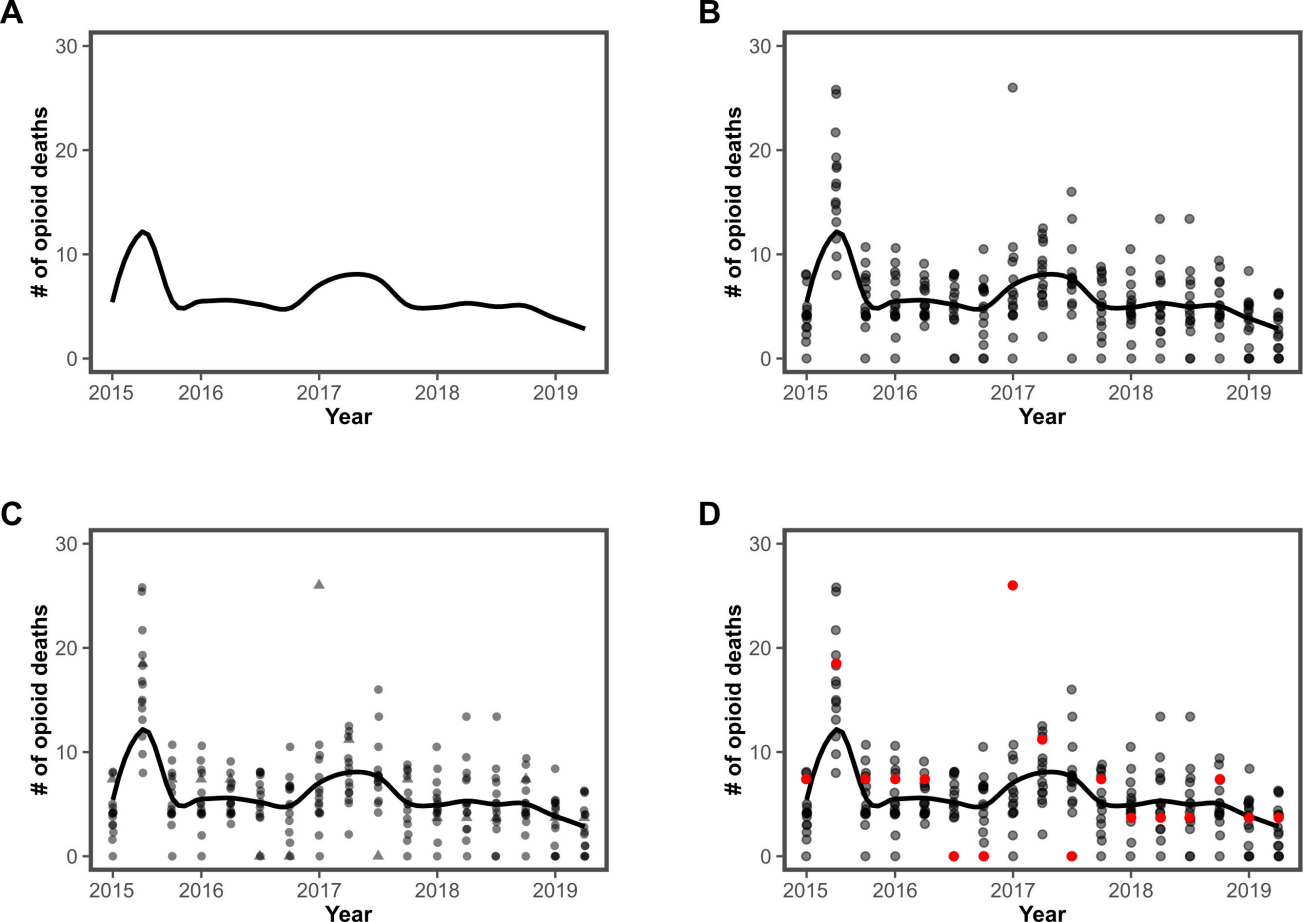
Scatterplot variations of the same synthetic data of opioid deaths versus time. (A) Panel A shows the general trend in opioid-related overdose deaths for all counties in an illustrative state. (B) Panel B adds markers for each county. (C) Panel C distinguishes a county of interest using a different shape. (D) Panel D distinguishes the same county of interest using color.

A well-designed dashboard allows end users to quickly extract key insights and draw meaningful inferences. Within the design community, a widely used approach is to identify and/or envision different user “personas”—distinct groups of individuals who will interact with the data—and tailor the visualization to meet their specific needs [16]. Effective visual design prioritizes clarity and accessibility. High-level, at-a-glance insights can be embedded in intuitive graphics that communicate key trends immediately, while more detailed or supporting information can be provided separately in text or tables that would take additional time to process [17]. Well-crafted data visualizations enable users to perceive both central tendencies and variability immediately. For instance, a straight trend line clearly indicates whether a pattern is increasing or decreasing over time. However, as illustrated with synthetic data in Figure 2A, a flexible line—such as a curve generated by mathematical smoothing techniques like beta splines or locally estimated scatterplot smoothing—can reveal nuanced departures from linear trends [18-20]. These features are particularly important for data that could have seasonal trends (eg, drug overdose patterns that vary by time of year) or identifying sudden shocks in data, such as unexpected spikes in mortality.

Combining multiple visualization elements enhances interpretability. As shown in Figure 2B, overlaying individual data points on a trend curve allows users to assess both the overall pattern and the underlying variability. This approach is especially valuable for identifying extreme events, such as a sudden surge in mortality due to drug supply contamination. At the same time, it helps distinguish genuine outliers from data anomalies that may stem from reporting errors rather than true shifts in the trend. When relevant, tagging outliers with contextual metadata can prevent misinterpretation (eg, a change in data reporting rather than a change in the underlying trend) and ensure that decision-making is based on accurate, reliable insights. By applying these visualization principles, dashboards can effectively support data-driven decision-making, enabling users to quickly grasp patterns, detect anomalies, and respond to public health challenges with confidence.

Some visualizations are static, meaning they function as fixed images where the information displayed remains unchanged. These are essentially bitmaps; what the user sees is exactly what they get. However, digital dashboards presented in a web browser or app can incorporate interactivity, allowing visualizations to change based on user actions. Interactive features enhance user engagement and data exploration. For example, users can zoom in or out on a graph by clicking buttons, dragging a slider, or pinching on a touchscreen. Filtering options, such as checkboxes, allow users to display or hide specific subgroups based on characteristics like race, age, or sex. Another valuable feature is the hover-over function, which reveals additional details when a user places the cursor over a data element, providing deeper insights without cluttering the initial visualization. While interactivity can significantly improve a dashboard’s functionality by allowing users to focus on information most relevant to them, it comes with trade-offs. Implementing interactive features requires additional human and computational resources, both for development and ongoing maintenance. Design challenges also emerge, as excessive interactivity can be distracting or even counterproductive. Too many visual elements, animations, or overly complex interactions may overwhelm the user, while poorly optimized interactivity can slow down rendering, diminishing the user experience. Thoughtful design ensures that interactivity enhances rather than hinders the dashboard’s effectiveness, striking a balance between flexibility and usability.

To ensure clarity and prevent misinterpretation, dashboard visualizations must be tested with multiple, diverse audiences. This is especially critical in public health, where data suppression is often necessary to protect anonymity or account for incomplete reporting. Misrepresentation can be particularly problematic when tracking rare events, such as overdoses. In small communities, just a few deaths can cause sharp fluctuations in rates, while in large urban areas, high death counts may appear less pronounced due to the sheer size of the population. In the HCS data dashboards, population-based rates were used to account for these disparate but real-world scenarios. However, even these metrics required careful handling. Overdose death rates, for example, were based on highly sensitive data that had to be suppressed in locations with low case counts to protect individual privacy. Simply omitting data points to comply with suppression rules could create misleading gaps in trend lines, making it difficult for policymakers to assess emerging patterns. Instead, smoothing techniques, such as locally estimated scatterplot smoothing curves (as shown in Figure 2A), allowed users to view approximate trends without revealing suppressed values, preserving both privacy and interpretability.

**Figure 3.**
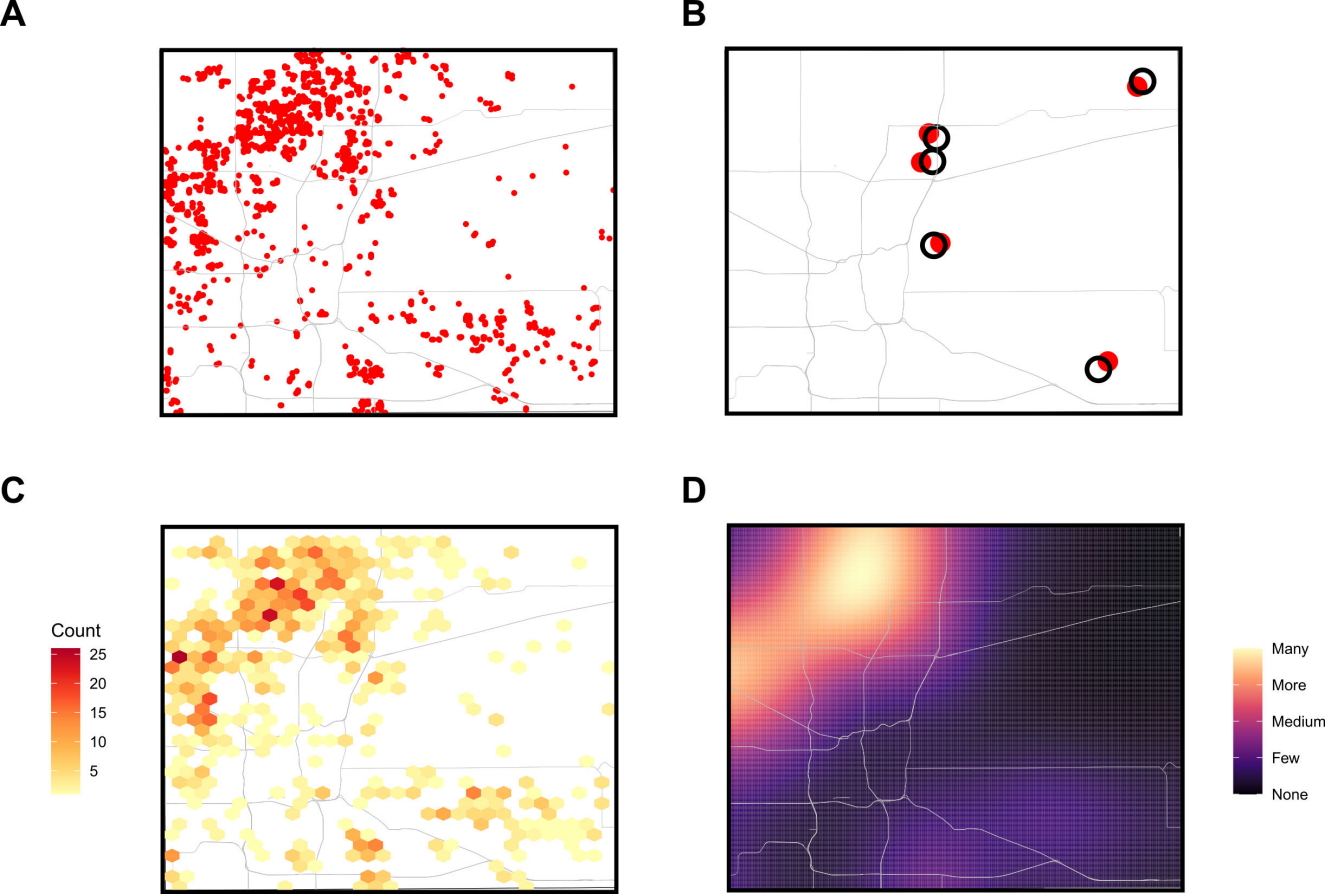
Map variations of the same geographical data. (A) Panel A shows an illustrative county with its primary roads along with simulated locations of overdoses, which are shown as red dots. (B) Panel B shows the same county with only 5 simulated overdose events displayed as large red dots along with slightly shifted (mean =1.25 km) locations as black circles. (C) Panel C shows the counts as colored hex shapes (hexagon area =1.4 km^2^) with deeper red colors indicating many events. (D) Panel D shows a density plot from kernel smoothing (40×40 km, max-min normalization) [21], with brighter and lighter colors indicating more overdoses.

Similar challenges arise when incorporating maps into dashboards. Policymakers rely on geospatial data to allocate resources—such as overdose reversal kits—to the areas most in need. However, to protect individual privacy, maps cannot display the precise locations of overdoses. Various methods can address this issue, each with trade-offs in accuracy and usability. One approach, illustrated with maps of synthetic data in Figure 3B, involves randomly shifting (“jittering”) [21], overdose locations to nearby points on the map; this preserves the overall count information but could foster misinterpretation for users who do not read the legend carefully. Alternative methods include overlaying a grid and shading areas based on case density or using mathematical density functions to blur exact locations. While these approaches help preserve anonymity, they can reduce granularity and make interpretation more challenging. Additionally, choices on the distance to offset or the amount of blur (Figure 3C and 3Ds) are complex [22,23]. A higher number of events in very densely populated areas (eg, urban areas) can safely allow for smaller offsets or blur, but a lower number of events in sparsely populated regions (eg, rural areas) requires larger jitter parameters to hide the exact locations of events.

Ultimately, the most effective visualization strategies depend on the needs of the end users. Engaging with stakeholders throughout the design process ensures that visualizations balance privacy, accuracy, and usability, making them both ethically responsible and practically useful for decision-making. Consistent with the community co-creation ethos described earlier, the HCS used the community-engaged data science model [15] as an approach to community co-creation key considerations, such as differences in data familiarity, interpretation, accessibility needs, and perceived relevance across roles, geographies, and communities. Community co-creation inputs informed visualization design decisions, including choices related to language and reading level, use of plain-language labels or multilingual resources, level of detail, and use of interactivity. Community engagement also informed decisions related to data equity (eg, when suppression risked obscuring inequities and how data gaps were annotated) and how uncertainty or data limitations were communicated. In addition, stakeholder input highlighted practical access considerations, such as the need to account for low-bandwidth environments, mobile access, and alternative formats for resource-limited settings. We note that approaches to community engagement are likely to vary across communities and over time, reflecting differences in local context, resources, intended dashboard use, and public health topics.

Consulting with graphic designers when building dashboards can greatly enhance the clarity and usability of data visualizations. Experts in visual design understand how to optimize information processing, making it easier and faster for end users to extract key insights [24,25]. Principles such as the Gestalt laws leverage the human tendency to group related elements based on similarity and proximity, allowing users to quickly interpret patterns and relationships within the data. The ideal visualization enables users to grasp critical information at a glance.

Reducing cognitive load is a core principle of good design. Directly labeling elements within a visualization, rather than relying on a separate key, prevents users from having to remember reference points while analyzing the graphic. Other mnemonic techniques, such as using meaningful symbols (eg, hollow dots to indicate missing or suppressed data), can further streamline comprehension. However, such symbols must be clearly defined to avoid confusion. Effective visual design requires continuous feedback from end users to ensure that all details are easily understood across diverse audiences.

Certain chart types are inherently more effective than others. Research has shown that bar charts and dot plots allow for faster and more accurate comparisons than pie charts, which can be more difficult to interpret [26-28]. In addition to choosing the right type of visualization, graphic designers use visual signals—such as color, shape, and size—to draw attention to key data points [17,29]. For instance, Figures 2C and 2D compare 2 methods for highlighting a county of interest, one using shape and the other using color. Determining which method is more effective depends on factors such as the overall size of the visualization, the density of the data, and the distribution of key elements. Direct engagement with end users can help refine these choices, ensuring that the most intuitive and efficient design is selected.

The design process should begin with an initial sketch or storyboard of the layout, progress through choices and prototypes, and culminate in a final, refined display. Dashboards are often accessed across devices with different screen sizes and bandwidth constraints (eg, desktop use in a networked office vs use on a personal mobile device with cellular bandwidth). Design decisions related to responsive layouts, content prioritization, and simplification of visual elements may therefore be needed to support use on smaller screens or in lower-bandwidth settings. These decisions may include determining which metrics or visualizations need to be retained across screen sizes and connection types, which elements can be condensed or hidden without loss of meaning, and how interactions are adjusted when space or network capacity is limited. Throughout this process, accessibility must remain a priority. Removing unnecessary clutter helps users focus on essential insights without distraction [30]. Additionally, dashboards should comply with applicable accessibility standards, such as the Americans with Disabilities Act (ADA), to support inclusive use. In practice, this may involve attention to issues such as color contrast and color blindness, keyboard navigation and focus states, the use of alternative text for non-text elements, and accessible labeling or markup to support screen readers. Addressing these considerations can help ensure that dashboards remain usable for individuals with a range of visual, motor, and cognitive needs. Accessibility checklists may be useful tools, including those informed by the Web Content Accessibility Guidelines (WCAG), for reviewing common accessibility considerations. Thoughtful, user-centered design ultimately ensures that dashboards are both functional and accessible, allowing all audiences to interact with data effectively.

In addition to the design decisions for the visualizations themselves, dashboards need to be constructed to allow users to expand their knowledge beyond what is on the page through proper attribution to the data. Data attribution refers to listing the data source for the underlying data used in the visualization. Specifically, the design team needs to decide if those details will always be visible, which increases visual clutter, or available on demand when users interact with the display, increasing the risk of users not identifying the sources of the data. For example, a visualization of drug overdose deaths may include a note that the data originate from the December 2022 mortality report from the state department of health. For the HCS displays, where we had a great deal of information to put on the dashboard, we typically opted to display additional information when the end user hovered over data or clicked on buttons to learn more. Feedback gathered from the community suggested that they were able to easily glean the details for the sources of the data.

### Hosting and Publishing Dashboards

Hosting and publishing dashboards are shaped by governance considerations that affect data access, release, and ongoing maintenance (Textbox 4). Rather than prescribing a specific governance model, this playbook emphasizes key governance-related decision points that arise as dashboards are hosted and published, such as who has authority to approve data use and release; how DUAs and privacy requirements (eg, 42 CFR Part 2 obligations) are monitored over time; how suppression rules are implemented within analytic and visualization workflows; how changes to data sources, metrics, or visualizations are reviewed and communicated; and how equity and inclusion goals are incorporated into these processes.

Textbox 4. **Decision points for hosting and publishing dashboards.**

The following questions highlight key considerations when planning the infrastructure, resources, and processes required to host and publish dashboards:

- What are the cyberinfrastructure requirements for data storage, creation of visualizations, publishing the dashboards, hosting the platform, and access permissions?
- What are the human resource requirements (eg, key roles, levels of expertise needed, and amount of effort)?
- Does this infrastructure meet requirements and regulatory rules for data storage and security?
- What are the existing institutional capacities with regard to servers, data visualization tools, and content management systems?
- What is the schedule for testing and quality assurance that will achieve a smooth publication of the official release version of the dashboard?

Implementing dashboards requires technical resources for both hosting the dashboard software and the data engineering processes necessary to convert raw source data into standardized measures [14]. Infrastructure requires resources to host or purchase commercial web hosting services. Personnel are needed for multiple roles and tasks, back-end database management staff, analytical staff to generate clean, standardized results, embedded testing staff for quality assurance, visual designers to implement effective visualizations, front-end engineers to produce dashboards and visualizations for end users, and a manager to coordinate internally and communicate externally to engage key stakeholders and the dashboard’s target audiences. Real-world resource constraints may dictate that more than one role and task be assumed by one individual. By some combination of skills and experience, technical expertise at every level of the process—as well as allocating sufficient effort and time—is just as integral to success as the infrastructure itself.

In addition to staffing and infrastructure needs, hosting and publishing dashboards involve ongoing resource and cost considerations beyond initial development. These can include the scale and complexity of data pipelines and hosting environments, recurring effort related to system administration, security reviews, software updates, and dependency maintenance, and the level of effort needed to support different refresh cadences or degrees of automation. As in the HCS, decisions about hosting and publishing often require balancing available staffing, technical capacity, desired functionality, and timeline considerations. Hosting and publishing dashboards also involve a range of operational tasks across build-out, launch, and maintenance. In practice, this may include clarifying staff roles and responsibilities for tasks, such as data engineer for extract, transform, and load processes; software engineer for visualization coding; designer for website design; and administration and support staff for hosting and administration, user support, and coordination across technical and programmatic staff. Operational considerations may also include determining appropriate data refresh patterns for different metrics and ensuring that routine maintenance activities—such as monitoring data pipelines, updating dependencies, and addressing security issues—are incorporated into ongoing workflows. Consistent with the case-based nature of this manuscript, these issues are treated as considerations rather than as a prescriptive costing or operational blueprint, with feasible approaches varying by organizational size, mission, context, and the maturity of available technologies.

The data infrastructure supporting raw data, analytical results, and dashboards must meet or exceed the security and protection requirements specified in the associated DUA and, when applicable, comply with broader regulatory frameworks, such as the HIPAA. For instance, storing and processing unsuppressed data may necessitate a secure network server with robust cybersecurity measures before the data are processed and prepared for publication.

Access control is another critical consideration. Depending on the sensitivity of the data, it may be necessary to restrict access to entire dashboards or specific features. Managing user access requires dedicated human resources for tasks such as account creation, access approval, validation, and ongoing monitoring. Access models can range from fully open dashboards, where no account management is needed, to tightly controlled environments in which each user must be individually verified and assigned an account. Intermediate approaches include self-managed accounts, where users can opt-in or sign up for access, balancing security with convenience. Integration with external authentication systems can further influence infrastructure requirements. For example, allowing Open Authorization—which enables users to log in with existing credentials from work accounts, Gmail (Google LLC), or other third-party services—can simplify access management but requires additional technical and security considerations. Ensuring that the cyberinfrastructure can support these access control mechanisms while maintaining compliance with security and privacy regulations is essential for safeguarding sensitive data while maintaining usability.

There is a wide variety of software for dashboarding, database management, analysis, and content management with varying degrees of cost and technical skill needed for implementation. Important considerations for dashboard development teams during evaluation, comparison, and selection of platforms and services include costs for licenses and feature sets; required technical expertise; scalability (eg, based on data volume, types of visualizations, user base size and heterogeneity, and accessibility); and the maintenance burden. Additional considerations include if and how dashboards can be embedded into other web content—for example, by providing application programming interfaces along with attendant restrictions on reproduction and further use—and how well tools integrate with existing infrastructure and workflows. In the HCS, different sites selected tools based on local expertise, resources, and institutional constraints. Open-source solutions, such as Shiny (Posit PBC) or D3, minimized licensing costs and allowed for greater customization but often required more advanced programming skills to implement and maintain. Commercial platforms, such as Tableau (Salesforce) and Microsoft Power BI, provided graphical user interfaces that supported rapid development of common visualization types but were less flexible for customization. In addition, academic and government organizations may already have enterprise licenses for commercial dashboard software, which can strongly influence platform selection.

Prior to publication and dashboard launch, the project implementation team should outline a plan for end user testing and review of all data visualizations, including feedback on navigation, design style, functionality, and processing speeds. Staging or test sites are common and widely available across different hosting platforms and should allow for different user access and permissions during this prelaunch review phase. Unforeseen obstacles are common and should be included in any work plan and accounted for in project launch timelines, as one programming solution will usually lead to a new and separate issue to solve. Often overlooked, testers need to also test for acceptability and performance on different device and browser types for any web-based application during a review phase. Once a thorough and transparent feedback and review process has been completed and final dashboard versions are ready for launch, it is recommended to use a “soft launch” to allow for time to address any bugs not detected on test site development. While every system and dashboard build varies and presents different challenges from development to publication, maintaining flexibility and the ability to adapt to new inputs or requirements is critical.

### Postlaunch Feedback and Revision

After the initial launch, live dashboards require routine maintenance and resolution of bugs to maintain trust and prevent burdensome obstacles to stakeholder engagement (Textbox 5). This requires a transparent mechanism in place for users to report bugs, submit feedback, and request new data or visualization formats. This mechanism should be easily accessible to end users either using an embedded form or a contact email address solely used for reporting issues or general feedback. Whichever method is implemented, site administrators would optimally receive automatic alerts to facilitate responsiveness. The dashboard team should also maintain a tracking tool to keep detailed records of each case to respond quicker and prevent similar issues in the future.

Textbox 5. **Decision points for postlaunch feedback and revisions of dashboards.**

The following questions highlight important considerations for monitoring dashboard performance after launch, collecting user feedback, and implementing revisions to improve functionality and usability over time.

- What is the plan for identifying bugs and issues and then implementing resolutions on an ongoing basis?
- What types and levels of end user feedback are integral to dashboard optimization, revision, and/or sunsetting?
- What are available methods for collecting end user feedback?
- What processes are in place for revising and enhancing dashboard components?
- How are postlaunch changes to metrics and visualizations documented and communicated to users?

Ongoing long-term feedback and revision of the dashboard system are equally necessary processes for assessing and evaluating whether dashboard aims are being met. These processes can iterate over the course of a project timeline but should be given consideration and built into initial scopes of work in early stages. Specific dashboard aims will differ depending on the project criteria; however, a central question in all public health dashboards should center on whether key metrics reach the intended audience and meet their needs. Are end users receiving useful information quick enough to inform decisions, understandable, and easy to apply in their own work or research? The answer to this question can determine whether information or insights gleaned from a data dashboard is in fact actionable in real-world settings (eg, setting program targets and monitoring implementation outcomes).

There are various methods to assess end-user experience. Well-documented and applied methods to collect feedback include usability testing (ie, direct observation of a participant performing a task), user surveys, focus groups, interviews, and website analytics tools that collect real-time site traffic data [31,32]. Each of these methods varies in resources required to implement and the types of data and insights they elicit. Balancing these factors and identifying one or a combination of methods throughout the course of the project can help ensure dashboard sustainability, as this feedback should be a key factor in the dashboard implementation team’s decisions on potential revisions and enhancements to the initial platform content and design.

Website analytics tools (eg, Google Analytics) require few resources to set up and provide useful real-time and trend data on user sessions, most visited pages, average time spent on a page, and pathways of navigation. A simple metric, such as which pages are most visited, should not be overlooked, as this can inform how to prioritize certain visualization enhancements and modifications over others. Website analytics also provide data on end user characteristics (eg, where users are located, device type, and language), which are important inputs when prioritizing revisions and new feature development.

User surveys are a common method for gathering website feedback and vary in resource allocation from automated pop-up surveys that require little maintenance to more comprehensive questionnaires that may incorporate validated usability and user satisfaction scales, such as the SUS or its modified version, the Standard User Experience Percentile Rank Questionnaire (SUPR-Q) [33]; these validated questionnaires include prompts that address end users’ attitudes about data credibility, appearance, ease of navigation, and willingness to return to the website and recommend to others. Despite the benefits of using these validated questionnaires, they alone will not provide the level of specificity needed to make targeted revisions for any single dashboard audience. While requiring more time in survey design, recruitment, and analysis, a more comprehensive survey that combines targeted prompts based on existing dashboard visualizations could prove to be a valuable source of end user feedback.

Focus groups, live webinars, and in-depth interviews are a more direct method of collecting end user feedback. While resource-intensive, these ensure direct community input and an opportunity to collect more nuanced real-time feedback from key stakeholders. In our prior usability study with the HCS, one important lesson we identified was the need to use an interdisciplinary approach to understand and improve engagement with data visualizations, which involves use of innovative technologies such as eye-tracking and audit log files and use of plan-do-study-act cycles to ensure fidelity with the co-creation process [12]. Such approaches generate multiperspective feedback that can support better design of a data dashboard.

Measuring a dashboard’s usefulness and ability to facilitate an action or decision is often a challenge for any dashboard evaluation. While the various feedback collection methods mentioned, combined with website analytics, may provide insight into end-user satisfaction and dashboard traffic, they will not sufficiently capture the breadth of how end users are applying information from the dashboard and insights learned in real-world settings. A routine process of soliciting these examples from key dashboard stakeholders may involve pop-up prompts, requests via email listserv, and invitations to participate in follow-up surveys, webinars, focus groups, or user story-telling events. The process of gathering examples of real-world applications of dashboard content should repeat over the lifetime of a dashboard, and sustained documentation of these occurrences is integral to a comprehensive evaluation at the end of a project cycle.

Evaluation of public health dashboards can occur at multiple levels, ranging from usability and user experience to patterns of use and, in some cases, downstream decision-making. As noted earlier, evaluation in the HCS focused primarily on usability and perceived usefulness, using the SUS and qualitative interviews informed by the Technology Adoption Model [12]. Beyond usability, teams may also benefit from examining how dashboards are used over time (eg, views, click-through rates, and dashboard item interactions) and how insights from dashboards inform decision-making processes. However, appropriate evaluation metrics, thresholds, and study designs (eg, AB testing) depend heavily on context, including dashboard purpose, data availability, organizational capacity, and intended use.

Once mechanisms for collecting evaluation data and feedback are in place, consistent and organized documentation of evaluation findings, end user feedback, ongoing maintenance issues, and new data and features requests is essential to informing prioritization. A collaborative process between dashboard implementers, key project stakeholders, and end users must weigh factors ranging from feasibility and required resources to potential impact when deciding which dashboard components to revise and enhance [34]. Across the HCS dashboards, feedback from different sources and endeavors informed multiple variations and versions of dashboard content and design. For example, qualitative feedback interviews indicated a need for support in data interpretation; this led to the team revising the dashboards to include “story” panels that contained brief textual summaries of temporal trends in the data. In another instance, community members raised questions during coalition planning meetings about the reach of online antistigma campaigns, which prompted the team to update the dashboard website to include visualizations of page views and click-through rates by campaign. These examples illustrate how user feedback informed dashboard development decisions without relying on a single, standardized feedback-to-change workflow.

As dashboards evolve post launch, changes to data sources, metrics, calculations, or visualizations should be tracked and documented (eg, changelog) in a systematic fashion. This includes situations in which metrics are modified, replaced, deprecated, or no longer supported due to changes or updates in data availability, definitions, reporting, or analytic approaches. Clear documentation of these changes can help maintain transparency, support interpretation of trends, and reduce confusion among end users, particularly when historical values are revised or when metrics are removed. While specific approaches to versioning, changelogs, or handling deprecated metrics will vary by platform and organizational context, the key task is considering how changes are documented and communicated.

## Discussion

### Summary

Well-designed dashboards serve as powerful tools for communicating public health data to key stakeholders and communities. Just as a carefully crafted automobile instrument panel can ensure a driver can reach their destination safely, an effective public health dashboard empowers community stakeholders to make informed, data-driven decisions. This is particularly crucial when epidemiological trends are rapidly changing or evolving across different locations and time periods. Unlike static data displays, interactive dashboards with real-time data provide a means of tracking and responding to public health challenges as they unfold. This playbook for developing data dashboards outlined steps and key considerations in data curation, design of visualizations, dashboard hosting and publishing, and feedback and revision.

### Limitations

While we expect this playbook to be valuable for a wide range of public health applications, our experience with the HCS may not always be generalizable. HCS focused specifically on opioid overdose and opioid use disorder, shaping the selection of dashboard metrics and data to reflect epidemiological trends and modifiable factors related to overdose prevention and treatment, such as safer prescribing practices and naloxone availability. The unique challenges encountered—such as suppression rules for sensitive data—may not directly apply to other public health issues, particularly those with different temporal, geographical, or population-level dynamics that influence the usability of visualizations and dashboard features. The intervention being tested in HCS was highly community-engaged; thus, opportunities for community co-creation and feedback for dashboards may have been more readily available. We also recognized that the underlying basis for this material is a series of case studies rather than a systematic, empirical investigation of dashboard development and deployment. The coordinated, multisite structure of the HCS allowed individual dashboard teams to benefit from cross-site discussion, problem-solving, and deliberation around key considerations. While the lessons presented here reflect consensus developed across multiple teams, similar opportunities for coordination and shared learning may not be available in other dashboard development efforts. Finally, as part of a grant-funded study, HCS dashboards were not required to include formal or coordinated sustainability planning. As a result, issues shaping sustainability (eg, funding, technical debt, knowledge transfer, and ongoing stakeholder engagement) were not systematically addressed. We posit that these considerations remain important for future dashboard sustainability efforts but are also highly context- and resource-dependent, thus prohibiting us from recommending universal sustainability templates, diagrams, or checklists. Altogether, while this case-based tutorial playbook offers practical considerations, further research and evaluation are needed to refine effective and practical practices for designing and implementing public health data dashboards across diverse contexts.

### Resources

The HCS dissemination efforts include a brief video entitled “How to Build Opioid Data Dashboards for Community Decision-Making: Best Practices from the HCS” [35]. This is a preview for a more in-depth, 5-session webinar for organizations interested in building public health data dashboards; webinar recordings and downloadable resources can be retrieved from the Data Dashboard Learning Collaborative Portal [36] (access provided upon request). The HCS also created a publicly accessible GitHub repository [37] with materials from a workshop organized along the following topics: (1) data acquisition and quality; (2) co-designing dashboards; (3) implementing and using dashboards in the community; (4) best practices for dashboard use; and (5) supporting the dashboard enterprise. The repository also includes a DUA template, synthetic data, and sample code to construct metrics (eg, apply suppression rules), and workbooks to visualize data from our HCS dashboards.

### Next Steps

Although the examples presented in this playbook are drawn from HCS dashboards developed for opioid overdose surveillance, we aimed to identify and describe decision points and considerations with potential portability to other public health issues. By emphasizing decision points rather than specific or prescriptive solutions, the tutorial playbook is intended to support adaptation across settings where data sources, governance structures, regulatory constraints, and available technologies may differ. We have provided Multimedia Appendix 1 to provide illustrative information and examples of using the playbook for building a dashboard for maternal mortality. However, public health encompasses a vast array of topics, and unforeseen challenges may arise when applying these principles to different issues beyond the scope of this work.

The scientific principles guiding the co-design of public health dashboards are still emerging and most likely will be subject to continual evolution, with many concepts remaining open to interpretation. This playbook provides a structured approach to addressing the challenges of designing rigorous, stakeholder-informed dashboards, offering key insights into the who, what, why, where, when, and how of dashboard development within the context of opioid use disorder and the overdose epidemic. This playbook’s design is a representation of first-hand experience and lessons learned while co-creating dashboards to communicate important public health information to community stakeholders, not a representation of scientific research such as an empirical review. While sustainability was mentioned, it remains an area that warrants further exploration; readers and developers are encouraged to seek successful models from government-funded endeavors, public-private collaborations, or community efforts. Additionally, some notable technological advances—such as machine learning (ML) and generative artificial intelligence (AI)—emerged during the period in which these dashboards were developed but were not implemented at the time (hence, outside the scope of this case-based tutorial playbook). Future public health dashboards may explore the value of such technologies for dashboarding, including forecasting trends, identifying emerging patterns, or supporting scenario-based planning [38]. Beyond analytic applications, AI tools have also been described as potential “design collaborators” that may augment the utility, effectiveness, and appeal of visualizations [39]. At the same time, ML and AI raise important challenges, including data privacy and governance considerations, the need for specialized technical expertise, and the risk of misinterpretation or overreliance on model outputs (eg, with spurious findings or hallucinations). Taking such care, ML and AI in public health dashboards remain potentially exciting and valuable areas for further investigation. Altogether, as the allied fields and technologies continue to evolve, dashboard development paradigms should be refined and evolve with allied fields—such as data science, graphic design, psychophysics, and cognitive sciences—to maximize the public health impact that dashboards can bring to the field.

## Supplementary material

10.2196/83157 Multimedia Appendix 1 An example of generalizing the dashboard tutorial playbook to maternal mortality.

## Abbreviations

ADA: Americans with Disabilities Act
AI: artificial intelligence
CDC: Centers for Disease Control and Prevention
DUA: data use agreement
HCS: HEALing Communities Study
HIPAA: Health Insurance Portability and Accountability Act
ML: machine learning
NIDA: National Institute on Drug Abuse
NIH: National Institutes of Health
SAMSHA: Substance Abuse and Mental Health Services Administration
SUPR-Q: Standard User Experience Percentile Rank Questionnaire
SUS: System Usability Survey
WCAG: Web Content Accessibility Guidelines
