# Supplementary material for: Building Public Health Data Dashboards: Tutorial Playbook
Source: JMIR Public Health Surveill. 2026 Apr 9;12:e83157. doi: 10.2196/83157 (PMC13065236; doi:10.2196/83157)
Supplement: Multimedia Appendix 1 [file publichealth-v12-e83157-s001.docx]

Supplemental Appendix: An Example of Generalizing the Dashboard Playbook to Maternal Mortality

The tutorial playbook describes our collective experience developing dashboards related to the opioid overdose crisis. Below, we provide a table that provide a compact worked example of taking the core considerations and illustrative decision points relevant for the case of building dashboards focused on maternal mortality.

These supplemental tables demonstrate how the dashboard development process generalizes across public health conditions. While maternal mortality shares similarities with opioid overdose (rare events, disparities known, etc.), the specific implementation details differ based on data characteristics (different temporal patterns for pregnancy-related timing), linkages required across systems, and differences in key stakeholder groups. We also caution readers to understand that this example is intended to be illustrative rather than comprehensive.

| Table S1: Core Consideration and Maternal Mortality Dashboards | |
| --- | --- |
| Consideration | Maternal Mortality Dashboard Application |
| Who | - Primary end-users:   - State maternal and child health program directors   - State and local mortality review committees   - Policymakers focused on maternal health   - Quality improvement headers from hospital systems - Secondary end-users:   - Clinical providers related to women’s health   - Public health epidemiologists   - Health equity advocates   - Community members and concerned citizens |
| What | - Key metrics/visualizations:   - Outcomes metrics:     - Maternal morbidity rate per capita     - Pregnancy-related deaths by timing (during pregnancy, during delivery, after delivery)     - Access to high-risk care   - Stratifications     - Race/ethnicity     - Geographic regions (rural vs urban)     - Insurance status     - Age groups |
| Why | - Primary objectives   - Monitor progress toward state/local maternal morbidity reduction goals   - Data-driven improvement in maternal healthcare   - Identify disparities in populations at risk - Dashboard as productivity tools:   - Real-time or near real-time surveillance vs delayed annual reports   - Interactive exploration of complex data   - Accessible visualizations for diverse stakeholders   - Ability to drill-down beyond state-level data |
| When | - Development timeline:   - Planning phase, 3-6 months (stakeholder engagement, data source identification, data use agreements)   - Initial development, 6-12 months   - Pilot testing with stakeholders, 2-3 months   - Full launch, 12-15 months after project initialization - Update frequency:   - Vital statistics data, monthly with 3 month lag   - Hospital discharge data, quarterly with 3 month lag   - Syndromic surveillance, daily with 2-3 day lag   - Medicaid claims, quarterly with 6 months lag |
| Where | - Access tiers:   - Public dashboard     - Hosted on state health department servers     - Displays aggregated, suppressed data only     - Available without login   - Limited-access dashboards     - Dashboards showing case-level details     - Requires authorized account with password     - Highly restricted access via encrypted website     - Multi-factor authentication     - Audit logging of access |
| How | - Resources and infrastructure   - Data sources     - Vital statistics – in collaboration with state vital records office     - Hospital discharge data – in collaboration with state department for public health     - Medicaid claims – in collaboration with state agency   - Technology stack     - Data warehouse (database, HIPAA compliance)     - ETL pipelines (data engineering and data integration tools)     - Authentication: OAuth 2.0 with multi-factor authentication enabled   - Team composition/Human Resources     - Project lead (informaticists or epidemiologist)     - Data engineer (1 FTE)     - Visualization engineer/designer (0.5 FTE)     - System administrators (0.5 FTE)     - Clinical subject matter expert (as needed)     - Community representatives (volunteer advisory board) |

| Table S2: Key Decision Points in the Dashboard Lifecycle for Maternal Mortality Dashboards | | |
| --- | --- | --- |
| Decision Consideration | Relevant Standards | Material Mortality Context |
| Data Suppression and Privacy | - HIPAA privacy rule - CD/NCHS statistical guidance on disclosure control - State/partner specific confidentiality statues dictating suppression rules - FAIR Principles | - For maternal mortality: - Suppress cells with < 11 deaths (CDC standard for rare events) - Geographic suppression: counties with < 20,000 women - Time period aggregation: 2 year rolling averages for small populations - Secondary suppression where needed to prevent disclosure from deduction using counts for different time periods (e.g. 1 month is suppressed but the yearly total is not) |
| Data Quality | - FAIR principles (reusable) - CDC data quality assessment dimensions - ALCOA principles | - For maternal mortality, - Display data quality indicators (e.g., percent missing) - Flag uncertain data (“Data is preliminary and subject to change”) - Document data limitations (“Pregnancy is underreported in death certificates according to …”) - Validate against published reports - Document and display data lags (“Vitals data is updated monthly with a 3 month lag” - Include notes on methods (ICD-10-CM codes used, case definitions) |
| Visual Design for Branding and Color Palettes | - Web Content Accessibility Guidelines (WCAG) 2.1 - Section 508 technical standards - State/institutional branding guidelines | - For maternal mortality: - Use institutional color palette that meets required contrast ratio - Acknowledge partners and data sources as needed by data use agreements - Test with advisor board members representing diverse backgrounds - Ensure colors are distinguishable for colorblind users - Sequential palette for maternal mortality rates (light to dark purple) - Diverging palette for disparity ratios relative to state average - Categorial palette for cause of death (max 6 categories, colorblind-safe) - Label “data suppressed” where suppression is needed to protect confidentiality |
| Interactivity | - Web Content Accessibility Guidelines (WCAG) 2.1 for keyboard accessibility | - For maternal mortality: - Hover tooltips show exact maternal mortality rates, year, numerator and denominators - Keyboard navigation for all filters (race, geography, time period) - Click to zoom maps without pinch gestures - Reset button to reset to default view - Export data to CSV (if user has permissions) |
| Access Control | - NIST - HIPAA security rule - OAuth 2.0 - State audit requirements | - For maternal mortality, - Public dashboard: no authentication - Limited access: multi-factor authentication login, session timeout after inactivity - Role definitions: public, limited, admin - Audit logs retained for 7 years (compliance with state retention data requirements) |
